# The Physiological Effects of Prolonged Strain

**Published:** 1907-07-20

**Authors:** 


### The Hospital

A Journal of

The Medical Sciences and Hospital Administration.

New Series. No. 17, Vol. I. [No. 1089, Vol. XLII.] SATURDAY, JULY 20, 1907.

### The Physiological Effects of Prolonged Strain.

Dr. L. P. Gibson, of Cowes, has made some interesting notes upon the condition of Mr. S. F. Edge, who succeeded in driving a car 1,581 miles 1,310 yards in twenty-four hours, at an average speed of 65 miles 1,594 yards per hour. Mr. Edge, it appears, is a teetotaller, does not smoke, and is in the habit of taking systematic exercise, although he has long hours of office work. He underwent no special course of training, and during the race he had fruit, with occasional drinks of cocoa and beef-tea, some chocolate and beef lozenges. He also took one grain of extract of chewing-gum made up with chewing gum every hour, but he had no solid food. When the race was over he had some peas, bread, and a drink of water. He was in bed and asleep within three hours of the finish, slept well all night, and was eating a good breakfast at nine o'clock next morning. The pulse, temperature, and respiration were normal, and he was none the worse for the extraordinary and exhausting strain through which he had passed. The temperature before starting on the race was 98.4° F. and pulse 74. At the end of the race the temperature was 100° F. and the pulse 70, with hardly any visible upstroke. The slowing seems to have been due to exhaustion, and the blood-pressure was low owing to vagus control. Before the ride a specimen of the blood showed a tuberculo-opsonic index of 0.85. Another specimen, taken directly after the race, gave 1.17 index, showing that the power of resistance to the tubercle bacillus had actually been increased.

These results confirm what has long been known, that a man in good health may work himself to a stage of exhaustion without ill result, for the physiological results of strain, whether mental or physical, are quickly recovered from in persons of ordinary health whether of body or mind. It is quite otherwise in those who are subjected to prolonged strain under unhealthy, or only partially healthy, conditions and postmen, curiously enough, are said to furnish numerous examples. Formerly they went their rounds on foot, both in town and country, with moderate loads, and the occupation was healthy but monotonous. The conditions are now completely changed, both in town and in country. In town the postman has to climb numberless stairs, for the system of living in flats has largely increased in every large town, whilst in the country he carries heavier loads, and for greater distances, but he does so by means of a cycle instead of walking, as used to be the custom. Statistics show that the postman's occupation is no longer so healthy as it once was. The men suffer from the increased strain which is put upon them by the new conditions of their work, and it is probable that they will have to be selected with much greater care than has hitherto been necessary, and that their physical condition will require more constant supervision. It is clear, therefore, that a strain which can be safely borne and easily recovered from by a healthy person in the prime of life, may be disastrous when it is repeated too frequently or when it is undertaken by those who are in any way unsound, whether mentally or bodily. It is in these cases that medical supervision is of the utmost importance. Examination at skilled hands made at regular intervals will easily detect the beginnings of overstrain; slight changes in the heart, the lungs, the kidneys, disturbed sleep, irritability of temper foreign to the general temperament of the person, and the want of elasticity, known as "slackness," will all put the medical examiner on the alert and warn him that the prolonged strain must be relieved, for it is beginning to tell upon the patient. A mere change of work will be sufficient in many cases, but it is more in accordance with the data of modern physiology to order a change of food. The remarkable results obtained by Dr. Chittenden show that everyone overeats himself, and that the energy of the tissues does not come, as is generally supposed, from the proteid constituents of the diet. His experiments were conducted in one case with a detachment of 20 men from the Hospital Corps of the Army, who were kept under military discipline. For a period of six months they were dieted, the amount of proteid food being gradually reduced until it became a minimum quantity. It was found that as the proteids were cut off the muscle tone and the muscular strength were remarkably increased. A second series of experiments were made with eight University athletes who were in constant practice and in the pink of condition when they began to change their diet. These gentlemen were submitted to experiments lasting for five months, during which the daily intake of proteid food was reduced by more than 50 per cent. Every individual, without exception, showed a decided gain in his muscular power as measured by strength tests, he suffered less from fatigue after vigorous muscular effort than formerly, and his mental faculties were correspondingly sharpened. These results, as coming from a physiologist of such first-rate importance as Professor Chittenden, are of great interest. He has no dietetic fads, and his conclusions are based wholly upon experiment. It appears, therefore, that the effects of prolonged strain are best met by change of work and reduction in proteid food. Mr. Edge has evidently arrived independently at a similar conclusion as to diet by the short cut of experience as opposed to experiment.

